# From Light Pulses to Selective Enhancement: Performance Analysis of Event-Based Object Detection Under Pulsed Automotive Headlight Illumination

**DOI:** 10.3390/s26092595

**Published:** 2026-04-22

**Authors:** Leonard Haensel, Torsten Bertram

**Affiliations:** 1 Research Institute for Automotive Lighting and Mechatronics (L-LAB), Rixbecker Str. 75, 59557 Lippstadt, Germany; 2 Institute of Control Theory and Systems Engineering (RST), Technical University Dortmund, Otto-Hahn-Str. 8, 44227 Dortmund, Germany; torsten.bertram@tu-dortmund.de

**Keywords:** event-based cameras, pulse-width modulation, automotive lighting systems, vulnerable road user detection, autonomous driving

## Abstract

Pulse-width-modulated (PWM) automotive headlights enhance nighttime event-based camera detection, yet systematic parameter optimization for vulnerable road user detection remains unexplored. This study evaluates PWM frequency, duty cycle, light distribution, ego-vehicle speed, and ambient lighting under European New Car Assessment Programme-inspired crossing scenarios for cyclist and pedestrian detection. Results establish performance ranging from substantial improvements to severe degradation relative to continuous illumination. Cyclist detection achieves robust performance with high-frequency modulation across light distributions, while low-frequency operation with low beam produces severe degradation through background noise accumulation. Pedestrian detection requires high beam with street lighting enabled; low beam universally fails regardless of modulation parameters. Limited parameter combinations achieve simultaneous improvements for both targets. Detection performs optimally on retroreflective surfaces, while low-reflectivity clothing limits capability, requiring target-specific optimization.

## 1. Introduction

Vulnerable road users including pedestrians and cyclists account for a disproportionate share of traffic fatalities, with nighttime conditions presenting particularly severe challenges for automotive perception systems [1,2]. Reduced ambient illumination, high-contrast ratios between illuminated and shadowed regions, and rapid relative motion between ego-vehicle and target combine to stress conventional frame-based camera systems operating at fixed frame rates [3]. These challenging conditions motivate investigation of alternative sensing modalities that exploit fundamentally different operating principles to achieve robust detection performance under adverse lighting scenarios.

Event-based cameras offer an alternative sensing modality by responding asynchronously to per-pixel brightness changes with microsecond temporal resolution and dynamic ranges exceeding 120 dB [4]. However, object detection requires accumulation of sparse event streams into dense pseudo-frame representations over fixed temporal windows, with standard approaches employing 50 ms intervals [5,6]. This accumulation requirement introduces latency that diminishes the temporal advantages of asynchronous sensing, limiting system responsiveness in safety-critical scenarios where rapid detection enables earlier intervention. Active illumination provided by vehicle headlights presents an opportunity to enhance event generation beyond passive sensing capabilities while addressing the accumulation time challenge. Modern automotive light-emitting diode (LED) systems increasingly employ pulse-width modulation (PWM) for thermal management and adaptive brightness control. PWM operation introduces periodic brightness fluctuations at frequencies typically ranging from 100 Hz to over 1000 Hz [7]. When such modulated illumination strikes objects in the forward field of view, each brightness transition triggers events on the event camera independent of object motion. This artificial event generation supplements motion-induced events, increasing pseudo-frame density within shorter accumulation windows [8]. The PWM parameters defining frequency and duty cycle jointly determine temporal characteristics of the induced brightness pattern, while event camera bias settings control sensor sensitivity to these brightness changes. The photoreceptor bandwidth bias determines maximum trackable modulation frequency, and contrast threshold biases define minimum brightness changes required to trigger events [9].

Our previous semi-static characterization identified optimal PWM headlight and event camera bias configurations that enable substantial accumulation time reduction while maintaining detection accuracy at 10 ms [10]. However, the controlled experimental setup with stationary vehicle position and fixed illumination geometry limits direct applicability to real-world scenarios. Dynamic driving conditions introduce ego-vehicle motion, continuously varying object distances, ambient street lighting, and environmental factors that may alter optimal parameter configurations or degrade detection performance. Systematic validation under realistic operating conditions is necessary before practical deployment in autonomous driving systems.

This work extends the semi-static parameter characterization to dynamic validation scenarios representative of real-world nighttime driving. Experiments were conducted at the Aldenhoven Testing Center Track, inspired by the European New Car Assessment Programme (Euro NCAP) Pedestrian and Cyclist Nearside test protocol [11,12]. During these tests, the instrumented vehicle traversed the crossing scenario at speeds of 30 km/h and 50 km/h. Building on the semi-static findings, measurements focus on the optimal 976 Hz frequency alongside 200 Hz to assess performance at lower frequencies representative of cost-optimized headlight systems deployed in production vehicles. Many automotive LED systems operate in the 200 Hz range due to thermal management constraints and driver circuit limitations, making this frequency point practically relevant for near-term deployment [7]. Both 70% and 90% duty cycles are evaluated under low beam and pseudo high beam distributions. The pseudo high beam configuration employs geometrically adjusted low beam positioning to achieve illumination intensities comparable to a standard high beam. Crucially, this setup eliminates the spatial interference patterns caused by asynchronous LED driver timing at a 70% duty cycle, which were observed during the semi-static characterization [10]. Street lighting conditions are systematically varied using certified luminaires configured to meet the NCAP photometric standards, enabling assessment of ambient illumination effects on detection performance. Event camera biases employ the optimal configurations identified in the semi-static study. Each experimental configuration is compared against a continuous illumination baseline to isolate PWM-specific performance contributions. Detection performance is assessed through Average Precision (AP) defined as the area under the Precision–Recall curve. This metric is evaluated individually for the ’Pedestrian’ and ’Cyclist’ classes to provide a specific characterization of detection reliability for each target type [13].

This work provides three contributions. First, a systematic evaluation of the parameter space under Euro NCAP-inspired crossing scenarios establishes performance characteristics across modulation frequencies, duty cycles, light distributions, and ambient lighting conditions. Second, analysis of optimal configurations reveals that only a limited subset of parameter combinations achieves simultaneous improvements in detection for the cyclist and pedestrian target, due to divergent surface retroreflectivity requirements. Third, the characterization of street lighting interactions demonstrates that ambient illumination enables spatially selective event generation while simultaneously elevating background noise levels that constrain absolute detection performance.

## 2. Related Work

### 2.1. Event Accumulation in Event-Based Object Detection

Event-based cameras represent a paradigm shift from conventional frame-based imaging by responding asynchronously to per-pixel brightness changes rather than capturing scenes at fixed temporal intervals. The operating principle provides microsecond temporal resolution, a dynamic range exceeding 120 dB, and data-driven acquisition, where the sensor output scales with scene dynamics. Gallego et al. provide a comprehensive survey of event-based vision fundamentals, detailing the contrast-detection mechanism, asynchronous pixel operations, and emerging applications across robotics, autonomous systems, and scientific instrumentation. The survey establishes event cameras as particularly suitable for scenarios involving rapid motion, extreme lighting variations, and high-speed tracking requirements that challenge conventional sensors [4].

Complementing these foundations, Kugele et al. investigate the fundamental question of how many events are necessary for reliable object detection on the 1 Megapixel dataset [14,15]. By systematically analyzing the correlation between event counts and detection accuracy, the work establishes a lower bound on event density. Results demonstrate that detection performance degrades as event counts decrease. Zhou and Jiang survey deep event-based detection methods, identify the accumulation time as a critical bottleneck limiting real-time performance, and emphasize the trade-off between pseudo-frame density and system latency [6]. These works highlight the importance of accumulation-time selection for pseudo-frame generation and demonstrate the inherent trade-off between temporal responsiveness and detection accuracy. While shorter accumulation improves latency and preserves fast dynamics, it can lead to overly sparse pseudo-frames and degraded features when event activity is low. Thus, in low-motion or poorly illuminated and low-contrast scenarios, sparse event distributions still necessitate longer accumulation windows to reach sufficient pseudo-frame density for robust feature extraction.

### 2.2. Accumulation Time Reduction Strategies

Accumulation time directly affects system responsiveness in autonomous driving applications, where rapid detection enables earlier collision-avoidance responses. Standard event-based detection approaches employ 50 ms windows to ensure sufficient event density across diverse scenarios [5,16,17]. This accumulation duration introduces latency comparable to or exceeding conventional cameras operating at 20 frames per second, diminishing the temporal advantages of asynchronous event generation. In a critical driving situation, every meter counts, emphasizing the safety implications of reducing latency. Recent research seeks to reduce accumulation time through architectural and algorithmic innovations that extract features from sparser pseudo-frame representations.

Fan et al. introduce DTSDNet, a Dense-to-Sparse-Detection network specifically designed to reduce accumulation time by a factor of five while almost maintaining detection accuracy [18]. The architecture utilizes an event temporal image representation that preserves both motion characteristics and temporal structure within accumulated windows. A dual-pathway aggregation module processes events through parallel dense and sparse branches, where the dense pathway extracts rich spatial features using conventional convolutional operations while the sparse pathway operates on efficient sparse representations. Cross-pathway fusion enables the sparse branch to leverage semantic information from the dense pathway without incurring full computational costs of dense processing. Experimental validation on the 1 Megapixel dataset demonstrates that DTSDNet achieves state-of-the-art detection performance while reducing accumulation time from 50 ms to 10 ms with minimal accuracy degradation [14]. Critically, the network exhibits superior robustness to varying object speeds compared to baseline approaches, maintaining consistent detection across the velocity ranges encountered in real-world driving scenarios. This work establishes that sophisticated representation design and multi-scale feature aggregation can substantially reduce accumulation time requirements even under passive illumination constraints.

Lu et al. extend accumulation time flexibility further through FlexEvent, which addresses detection at arbitrary operational frequencies ranging from 20 Hz to 180 Hz [19]. The network introduces two key innovations enabling frequency-adaptive operation. First, the FlexFuse module integrates high-frequency event data with semantic information from synchronized Red Green Blue (RGB) frames through learned fusion strategies that adapt weighting based on instantaneous event rates. This hybrid approach leverages the complementary strengths of dense RGB features and sparse temporal event information, maintaining robust detection across scenarios where one modality dominates. Second, FlexTune generates frequency-adjusted training labels by analyzing ground truth annotations across different temporal resolutions, enabling the network to generalize effectively to operational frequencies not encountered during training. Experimental validation demonstrates that FlexEvent sustains detection accuracy when scaling from 20 Hz baseline performance up to 90 Hz, and maintains effective detection even at 180 Hz where conventional fixed-frequency approaches degrade substantially. The network proves particularly valuable for scenarios combining fast-moving objects requiring high temporal resolution with static or slow-moving targets where lower frequencies suffice. By explicitly parameterizing operational frequency, FlexEvent enables runtime adaptation to computational constraints and scene dynamics, advancing practical deployment of event-based detection in resource-constrained automotive systems.

These accumulation time reduction strategies achieve substantial progress through sophisticated network designs that extract features efficiently from sparse inputs and adapt to varying temporal characteristics. However, all approaches operate under passive illumination conditions where event generation depends solely on object motion and ambient brightness changes. The fundamental constraint remains that in low-motion scenarios or under poor illumination, even optimized architectures require longer accumulation windows to achieve adequate pseudo-frame density. No prior work has explored controlled event generation enhancement through active illumination modulation as a complementary strategy for achieving reduced accumulation times. The potential for artificial event generation using tailored illumination remains unexplored in automotive nighttime scenarios. Modern vehicle headlights offer the capability to create structured brightness patterns, which could increase event rates and enable architectural approaches to operate at significantly shorter accumulation windows [8].

### 2.3. Research Gap and Motivation

Our previous semi-static characterization provided a foundational understanding of parameter interactions between PWM headlight configuration and event camera biases for nighttime object detection [10]. The investigation employed pedestrian and cyclist targets positioned 30 m from a stationary vehicle within a controlled environment. Experiments tested four PWM frequencies (104 Hz, 200 Hz, 780 Hz, 976 Hz), duty cycles (30%, 50%, 70%, 90% 100%), and key bias parameters such as photoreceptor bandwidth and contrast thresholds. Three experimental phases isolated frequency effects, bias interactions, and duty cycle optimization sequentially.

The first phase characterized frequency dependence, revealing that 976 Hz achieved optimal detection performance while lower frequencies exhibited degraded accuracy. The 780 Hz and 976 Hz frequencies achieved cyclist AP of 0.94 and pedestrian AP of 0.90, while 200 Hz yielded cyclist AP of 0.96 and pedestrian AP of 0.89. These results indicated that frequency effects saturate above 780 Hz for both target types when ego-vehicle motion is absent. Due to the marginal differences in semi-static conditions, the dynamic validation examines frequency extremes (200 Hz and 976 Hz) to assess how vehicle motion, detection distance, and ambient light affect frequency-dependent performance in realistic driving.

In the second phase, we explored the joint parameter space of the photoreceptor bandwidth bias bias_fo, contrast threshold biases bias_diff_on, and bias_diff_off, identifying optimal configurations that maximize event generation on illuminated targets while maintaining event polarity distributions consistent with detection network training data and reduce environmental noise. Results demonstrated that bias_fo of 25 provides sufficient bandwidth for 976 Hz PWM, while bias_diff_on of 0 and bias_diff_off of 20 balance sensitivity against noise. The third phase evaluated duty cycle influence, establishing that a 90% duty cycle achieves peak detection performance by maximizing average illumination intensity while maintaining sufficient PWM-induced brightness transitions for artificial event generation.

The optimal configuration achieved an AP of 0.970 for cyclists and 0.920 for pedestrians at 10 ms accumulation time, exceeding continuous illumination baselines by 0.16 and 0.14 AP, respectively. These results validated that controlled brightness modulation provides detection benefits beyond illumination intensity alone, with artificial event generation enabling substantial accumulation time reduction compared to passive sensing. The investigation established that neither illumination configuration nor sensor parameters independently achieve optimal performance, demonstrating that joint parameter optimization is essential for maximizing detection accuracy.

However, the semi-static experimental configuration imposes significant limitations on practical applicability. The stationary vehicle position eliminates ego-motion effects on detection performance, while the controlled 11-meter trajectory with fixed 5 km/h dummy movement maintains constant object distance and approach geometry throughout each measurement. The HELLA test environment provides complete isolation from ambient illumination variability, preventing assessment of street lighting interactions or environmental brightness interference with PWM-enhanced detection. These controlled conditions enabled precise parameter isolation but limited generalizability to real-world nighttime driving.

Dynamic driving scenarios introduce multiple confounding factors that may alter optimal parameter configurations or degrade detection performance. Ego-vehicle motion creates continuously varying object distances and relative velocities that stress detection range and temporal stability. Street lighting contributes to ambient illumination that may interfere with PWM-induced event patterns or alter signal-to-noise characteristics. Environmental diversity, including varying weather conditions, road surface reflectivity, and oncoming vehicle headlights, presents challenges absent from controlled laboratory experiments. Most critically, the semi-static setup provides no validation that optimized parameters transfer to realistic operating conditions where autonomous systems must perform reliably.

As far as we know, no prior work has validated event-based detection performance under dynamic conditions combining ego-vehicle motion with controlled PWM illumination optimization. Existing accumulation time reduction strategies operate under passive illumination without exploring active enhancement approaches. Bias tuning methodologies focus on artifact suppression rather than signal optimization for artificial event generation. The gap between controlled parameter characterization and realistic deployment validation represents a critical barrier to practical implementation of active illumination strategies in autonomous nighttime driving systems.

This work addresses the validation gap by extending semi-static parameter characterization to dynamic test scenarios representative of real-world vulnerable road user encounters. Experiments employ Euro NCAP-inspired crossing protocols with moving ego-vehicle and varying speeds, assess robustness across street lighting conditions, and evaluate parameter transferability to realistic driving dynamics. The investigation establishes whether joint PWM and bias optimization identified through controlled experiments provides robust detection enhancement under practical operating conditions.

## 3. Materials and Methods

### 3.1. Experimental Setup and Instrumentation

**Figure 1:**
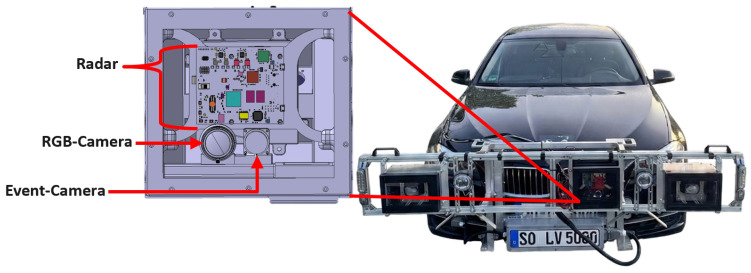
Close-up of the mounted sensor housing on the test vehicle. The enclosure contains a multi-sensor setup consisting of a radar, a RGB-camera, and an event-camera. The red arrows and brackets indicate the components inside the sensor housing.

The experimental platform employs a test vehicle instrumented with a multi-modal sensor suite for comprehensive data acquisition under dynamic driving conditions. The sensor configuration integrates event-based vision, conventional imaging, Radio Detection and Ranging (Radar), inertial measurement, and vehicle state monitoring. Event-based vision is provided by a Prophesee IMX636 evaluation kit camera with a 1280-by-720-pixel resolution (Prophesee, Paris, France). The sensor housing is mounted near the headlight module using a special carrier and is shown in Figure 1. This mounting location enables investigation of a potential sensor integration into the headlight assembly itself, exploring whether the reduced distance to the illumination source provide detection advantages compared to conventional windshield-mounted configurations. The mounting position provides an unobstructed forward field of view encompassing the test trajectory crossing zone while enabling direct assessment of headlight-integrated sensor architectures for future production implementations.

Ground truth object detection labels are generated using a Baumer VCXU-23C color camera (Baumer, Frauenfeld, Switzerland) with 1920-by-1200-pixel resolution operating at 50 frames per second. Precise spatial alignment and temporal synchronization between the RGB reference camera and event camera enables accurate bounding box reprojection for evaluation. Distance and velocity measurements are obtained from an IWR1843BOOST frequency-modulated continuous-wave radar (Texas Instruments, Dallas, TX, USA) operating at 77 GHz with an output rate of 10 Hz. The radar provides a range resolution of 0.04 m and a velocity resolution of 0.1 m/s. Vehicle dynamics are monitored through two complementary systems. An internal Measurement Unit (IMU) (RaceBox, Altamonte Springs, FL, USA) positioned on the windshield records three-axis acceleration and angular velocity at 10 Hz. The test vehicle’s controller area network (CAN) provides timestamped telemetry at 10 Hz including GPS coordinates, wheel speeds for all four corners, yaw rate, longitudinal and lateral acceleration, indicated vehicle speed, and engine rotational speed. The active lighting system uses a specially manufactured, PWM-controlled LED headlight module that has been mounted in front of the vehicle using a special mounting structure. The headlight system enables specific configuration of PWM frequency and duty cycle while maintaining Economic Commission for Europe (ECE) regulatory compliance for light distributions.

### 3.2. Test Scenarios and Protocol

**Figure 2:**
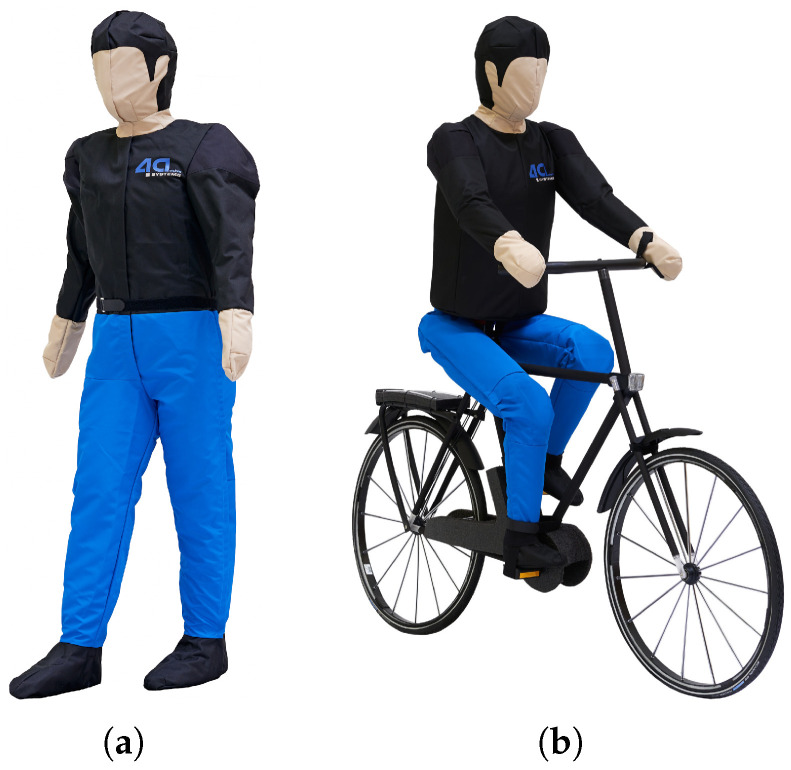
The NCAP-specified dummies utilized as targets [20]: (**a**) pedestrian and (**b**) cyclist.

Experimental validation employs crossing scenarios inspired by the Euro NCAP Pedestrian and Cyclist Nearside test protocols. The Euro NCAP methodology defines standardized trajectories for vulnerable road user testing in autonomous emergency braking evaluations, providing reproducible geometric configurations representative of real-world collision scenarios [11]. The test implementation adapts these trajectories for detection performance characterization rather than collision mitigation assessment, substituting the protocol’s impact speeds with reduced velocities suitable for repeated non-destructive measurements. Tests are conducted at the Aldenhoven Testing Center in Germany, which provides controlled outdoor environments with flexible infrastructure for manipulating lighting conditions. The facility features straight approach sections exceeding 260 m in length, enabling stable vehicle speeds prior to target encounter. Road surface characteristics match public road standards, and the testing area permits the installation of temporary street lighting equipment for ambient illumination studies. The crossing scenario geometry follows the Euro NCAP Nearside configuration where the target traverses the vehicle’s path from the passenger side toward the driver side at a perpendicular approach angle. The target initiates crossing motion from a standstill position offset from the vehicle trajectory, accelerating to constant crossing velocity as the vehicle approaches. Targets consist of purpose-built NCAP-certified test dummies (4activeSystems GmbH, Traboch, Austria) representing pedestrian and cyclist configurations, depicted in Figure 2 [20].

The pedestrian dummy features anthropomorphic dimensions and clothing representative of typical nighttime attire. In addition, the dummy has motorized leg movement to create the most realistic appearance possible. The cyclist dummy incorporates a bicycle frame equipped with retroreflective elements matching regulatory requirements for bicycle visibility equipment. Detailed specifications regarding target dimensions, clothing color properties, and retroreflective characteristics are provided in the referenced standards and target specifications [21,22,23]. Both dummies are mounted on motorized platforms that execute the crossing trajectory under computer control, ensuring repeatable crossing velocities and timing precision the measurement campaign. The dummy movement is initiated using a light barrier control system. This system also simultaneously monitors the speed of the ego-vehicle to detect measurement errors in time if the target speed is not reached or is exceeded.

**Figure 3:**
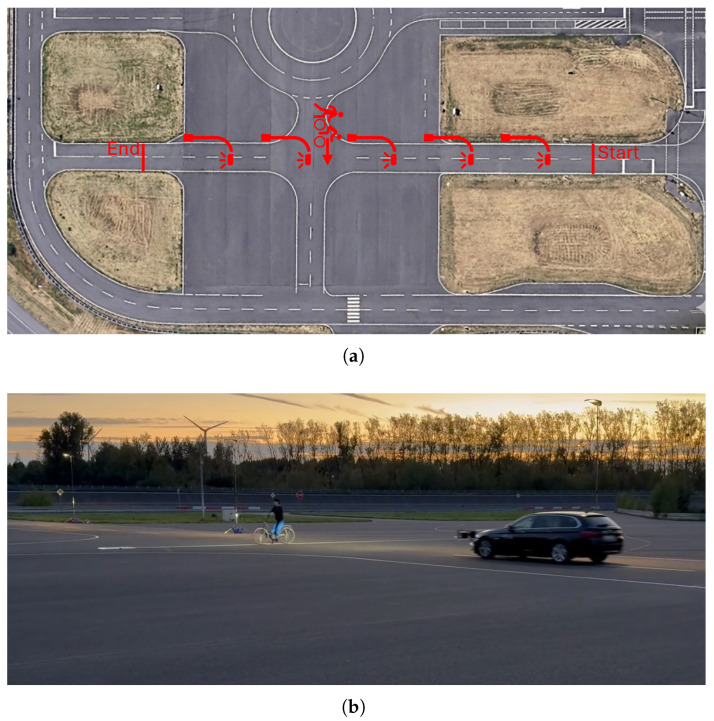
(**a**) Satellite view of the Aldenhoven Testing Center, specifically Urban Environment B. The overlay illustrates the experimental setup used for this study, with the “Start” and “End” lines marking the beginning and termination of the measurement interval. (**b**) Visualization of the test scenario featuring the vehicle under test and the cyclist dummy. Note that this image illustrates a trial run with high ambient brightness; the study data was recorded in darker environments.

For pedestrian scenarios, crossing velocity is maintained at 5 km/h, representing typical adult walking speed. Cyclist scenarios employ 15 km/h crossing velocity, reflecting moderate cycling speeds in urban environments. These target velocities combine with ego-vehicle motion to create relative approach dynamics representative of real nighttime encounters. Ego-vehicle speeds are systematically varied at 30 km/h and 50 km/h. The 30 km/h condition represents urban residential speeds where vulnerable road user encounters are frequent. The 50 km/h condition reflects arterial road speeds where higher approach velocities stress detection range and temporal response requirements. Each test run begins with the vehicle approaching from a distance exceeding 100 m, accelerating to target speed, and maintaining constant velocity through the crossing zone. Vehicle speed stability is verified through a light barrier, CAN bus telemetry and GPS velocity measurements, ensuring consistent experimental conditions across repeated trials. To give a better impression of the procedure, the measurement setup is shown in Figure 3.

Street lighting conditions are systematically varied between the presence and absence of ambient illumination. When activated, LED street lighting employs certified luminaires configured to meet NCAP photometric standards for residential road lighting without PWM-modulation [11,24]. Luminaire positioning and aiming angles are adjusted to achieve the specified average illumination levels on the road surface within the crossing zone, verified through photometric measurements before and after the entire measurement campaign. The controlled lighting environment enables isolation of ambient illumination effects on PWM-enhanced detection performance, distinguishing between scenarios where vehicle headlights provide sole illumination versus cases where street lighting contributes additional background brightness, which leads to additional noise in the event camera.

### 3.3. Active Illumination Configuration

The headlight illumination employs two PWM frequencies selected to span the range of practical automotive implementations [7]. The 976 Hz frequency represents the optimal configuration identified in semi-static parameter characterization, providing high temporal bandwidth for rapid brightness transitions. The 200 Hz frequency represents lower-cost headlight systems commonly deployed in production vehicles, where thermal management constraints necessitate reduced modulation rates. This frequency selection enables assessment of detection performance across the spectrum of likely deployment scenarios, from optimized research systems to cost-constrained production implementations. The duty cycle is varied between 70% and 90% to evaluate trade-offs between illumination efficiency and detection performance. The 90% duty cycle provides near-continuous illumination with minimal temporal gaps in brightness, maximizing average illumination intensity while maintaining sufficient PWM-induced brightness transitions to generate artificial events. The 70% duty cycle reduces average illumination intensity but increases the magnitude of brightness transitions between ON and OFF states, potentially enhancing event generation through larger contrast steps. The light distribution employs both standard low beam geometry and a pseudo high beam configuration. The low beam provides the asymmetric horizontal cutoff distribution required for a glare-free operation in traffic, concentrating illumination on the road surface ahead while limiting upward light projection [25,26]. The pseudo high beam configuration is achieved through mechanical adjustment of the low beam module’s mounting angle, rotating the optical axis upward to increase the range of the illuminated region. The geometric adjustment eliminates the asynchronous LED driver timing present in production high beam implementations, avoiding the temporal phase differences between independent LED driver circuits that cause spatial interference patterns at reduced duty cycles [10]. The adjustment procedure employed a mobile screen positioned 10 m from the vehicle, where the cutoff line of a reference high beam distribution was marked. The low beam module mounting angle was adjusted vertically until the projected pattern approximately matched the reference geometry, verified through visual comparison of cutoff line position. The upward rotation achieves extended detection range but reduces near-field illumination intensity directly ahead of the vehicle. This near-field reduction affects primarily the road surface directly ahead of the vehicle rather than vulnerable road users themselves, whose vertical extent ensures adequate illumination from the elevated beam at detection-relevant distances. Illumination intensity distributions for the low beam configurations are characterized through photometric measurements, verifying compliance with ECE regulatory requirements and enabling illumination-normalized performance comparisons. Each illumination configuration is measured alongside a continuous illumination baseline, with PWM disabled and LEDs operating at constant brightness. The continuous mode provides a constant 100% intensity baseline, enabling a direct comparison of detection performance with and without the artificial event generation characteristic of the different duty cycles. This baseline measurement strategy isolates the contribution of PWM-induced brightness transitions from general illumination intensity effects.

### 3.4. Data Acquisition and Synchronization

Data acquisition integrates multiple sensor modalities through a custom Python 3.9 software employing manufacturer-provided software development kits (SDK) for each hardware component. The system architecture manages simultaneous high-bandwidth data streams from the event camera, the RGB camera, the Radar, and the vehicle telemetry, storing all data streams with hardware-synchronized timestamps for post-processing analysis. The event camera interface utilizes the Prophesee Metavision SDK 4.6.2 to configure the IMX636 sensor with specified bias parameters and establish a continuous event stream to the acquisition computer. Events are timestamped at the sensor level with microsecond precision, capturing per-pixel brightness changes asynchronously as they occur. The SDK buffers events in system memory and writes them to disk in batches, preserving temporal order while minimizing acquisition latency. Bias parameters including bias_fo=25, bias_diff_on=0, and bias_diff_off=20 are programmable configured based on our last experimental results [10]. The RGB camera interface employs the Baumer GAPI SDK 1.7.1 to capture frames at 50 Hz with exposure settings optimized for nighttime conditions. Frame acquisition timestamps are recorded at the moment of exposure initiation rather than readout completion, providing an accurate temporal reference for synchronization with asynchronous event data. Exposure time and gain settings are fixed across all measurements to maintain consistent image characteristics, with values selected to avoid saturation from headlight illumination while capturing sufficient detail in shadow regions for ground truth annotation. Radar data acquisition is performed using the Texas Instruments mmWave SDK 4.7.0 to retrieve range-Doppler point clouds at 10 Hz. Each point cloud contains range, azimuth, elevation, and radial velocity measurements for all detected targets within the radar field of view. The SDK provides raw point cloud data, including the radar-cross-section (RCS) value. Radial velocity measurements enable verification of consistent target approach speeds across test runs, while range measurements provide distance ground truth. Point clouds are stored with acquisition timestamps synchronized to the hardware trigger system for subsequent projection into image coordinates during ground truth generation.

Vehicle telemetry from the controller area network (CAN) is logged through an external CAN-to-Universal Serial Bus (USB) adapter operating at 10 Hz. The adapter queries multiple CAN message identifiers simultaneously, recording GPS coordinates, wheel speeds for all four corners, yaw rate, longitudinal and lateral acceleration, indicated vehicle speed, and engine rotational speed. All parameters are logged with a single timestamp per acquisition cycle to preserve temporal relationships between vehicle state variables. This comprehensive telemetry monitors instantaneous driving dynamics to verify consistent test execution parameters, specifically approach speed stability across repeated measurements. In addition, engine speed is recorded to detect potential fluctuations in headlight brightness caused by the vehicle’s power supply. The RaceBox IMU records three-axis acceleration and angular velocity measurements internally. The IMU data is timestamped using the GPS clock and synchronized with the other sensor streams via post-processing.

The hardware synchronization of all sensor modalities is implemented through an Arduino microcontroller programmed to generate a 50 Hz periodic trigger signal. The microcontroller outputs separate digital pulses to all acquisition modules via dedicated output channels, ensuring simultaneous trigger delivery across all sensors. The trigger signal initiates a frame capture on the RGB camera, generating a hardware-timed exposure start that serves as the temporal reference for the measurement sequence. Simultaneously, the event camera SDK records trigger timestamps within the event stream, enabling precise alignment of asynchronous events with synchronous frame captures. Radar acquisitions are triggered at 10 Hz, providing range-velocity measurements temporally aligned with every 5th RGB frame. Vehicle telemetry data streams record trigger timestamps through software, establishing temporal correspondence with hardware-triggered sensors.

Post-processing algorithms use these hardware-trigger timestamps to establish frame-accurate temporal alignment across all data streams. For each RGB frame, the nearest event accumulation window is identified based on trigger timestamps, ensuring that the pseudo-frame is generated from events captured during the corresponding frame exposure interval. Additionally, we use linear interpolation of bounding box positions between consecutive frames to account for target motion during the accumulation window of 10 ms, providing a more accurate spatial reference for detection evaluation [3]. Radar point clouds are associated with frames using the same trigger-based matching, enabling the projection of range-velocity measurements into image coordinates for ground-truth validation. This synchronized multi-modal acquisition architecture enables comprehensive characterization of detection performance across varying experimental conditions while maintaining the temporal consistency necessary for accurate evaluation metrics. The hardware trigger system ensures sub-millisecond synchronization accuracy between heterogeneous sensor interfaces, eliminating temporal misalignment artifacts in ground truth generation and detection assessment.

### 3.5. Ground Truth Generation and Labeling

Object detection ground truth is established through a multi-stage process combining manual annotation and automated geometric projection. Initial bounding box labels are created through manual annotation of RGB camera imagery using the Roboflow Computer Vision Annotation Tool, where annotators mark pedestrian and cyclist instances with rectangular bounding boxes encompassing the full visible extent of each target [27]. Annotation guidelines specify that boxes should tightly bound the target with minimal background inclusion while ensuring complete coverage of extremities and bicycle components. Geometric transformation of annotations from RGB camera coordinates to event camera coordinates employs camera calibration parameters obtained through an extrinsic calibration with a reprojection error of 0.5 pixels. Intrinsic calibration determines focal length, principal point, and lens distortion coefficients for each camera independently. Extrinsic calibration establishes the three-dimensional rotation and translation relating the two camera coordinate systems through simultaneous observation of calibration targets visible to both sensors. The combined intrinsic and extrinsic parameters enable reprojection of RGB bounding boxes into event camera pixel coordinates, accounting for differences in field of view, resolution, and perspective between the two sensors. Radar measurements contribute additional ground truth information through automated target association. Radar point clouds are projected into RGB image coordinates using the extrinsic calibration between radar and camera coordinate systems. For each manually annotated bounding box in a given frame, the radar points falling within the box boundaries are identified as associated with that target instance. Range and radial velocity values from associated radar points provide distance and approach speed measurements for each detected object throughout the measurement sequence. Multiple radar points may associate with a single target in cases where the radar resolution cell is smaller than the target extent, in which case median range and velocity values are computed to provide robust estimates. This combined annotation approach provides comprehensive ground truth encompassing object location, extent, distance, and velocity for each detection instance. The multi-modal ground truth enables the evaluation of detection performance as a function of range and approach dynamics while maintaining pixel-accurate localization reference through the reprojected bounding boxes.

### 3.6. Object Detection Network

Object detection is performed using the YOLOv8 architecture implemented through the eTraM event-based object detection network [28]. The network operates on pseudo-frame representations generated by accumulating events into fixed-duration time windows. For each evaluation, events occurring within a 10 ms accumulation interval are aggregated into a two-channel image representation where one channel encodes positive polarity events and the other channel encodes negative polarity events. This representation maintains polarity information critical for feature extraction while providing a format compatible with convolutional architectures trained on conventional imagery. The detection network weights are initialized from training on the Stereo Event Camera Dataset for Driving Scenarios (DSEC), a large-scale event camera dataset collected from automotive scenarios including diverse lighting conditions and target types [29]. Training employed pedestrian and cyclist classes with bounding box annotations derived from synchronized conventional camera imagery. The DSEC training provided the network with exposure to event patterns generated under natural driving conditions, establishing baseline feature representations applicable to real-world scenarios. The network originally trained using an event accumulation of 50 ms. This configuration allows us to investigate whether the increased event density produced by PWM illumination can compensate for the shorter accumulation window and to what extent competitive detection performance can be maintained despite the reduced accumulation time. The network weights are frozen during evaluation on the experimental dataset, ensuring no fine-tuning or retraining is performed using the test measurements. This frozen weight approach ensures that performance metrics reflect the network’s ability to generalize from training data to new scenarios rather than adapting to dataset-specific characteristics. The decision to freeze weights mirrors practical deployment conditions where networks must perform on previously unseen data without opportunity for scenario-specific optimization.

## 4. Results

### 4.1. Experimental Consistency

**Figure 4:**
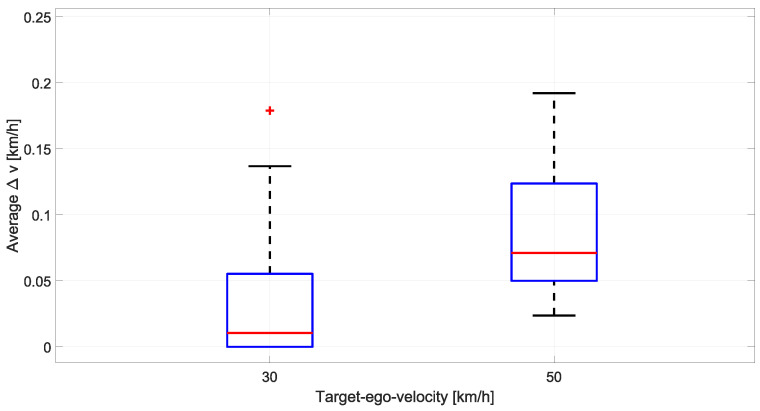
Ego-vehicle speed deviation (Average Δv) distributions for 30 km/h and 50 km/h test conditions. In the box plots, the solid red line indicates the median, the blue box the quartiles, black dashed lines the extreme values, and red pluses (+) outliers.

Systematic comparison of detection performance across 64 parameter and 16 reference measurements requires consistent experimental conditions to ensure that the observed differences reflect parameter variations rather than uncontrolled environmental factors or trajectory execution errors. Vehicle dynamics and target motion characteristics were monitored throughout all measurement campaigns to verify repeatability and enable fair comparison across headlight configurations and ambient lighting conditions. The Ego-vehicle speed stability was assessed through the limiter function of the cruise control system. Additionally, the CAN bus telemetry recorded throughout each measurement sequence. Figure 4 presents speed deviation distributions for both test conditions across all measurements. The 30 km/h condition achieved median speed deviation of 0.011 km/h with 75th percentile deviation of 0.055 km/h, representing coefficient of variation below 0.2%. The 50 km/h condition maintained median deviation of 0.071 km/h with 75th percentile of 0.124 km/h, corresponding to coefficient of variation below 0.25%. Maximum observed deviations remained below 0.2 km/h for both conditions, confirming that approach velocity stability was maintained throughout the crossing zone where detection performance was evaluated. The minimal speed variability validates that differences in detection metrics across experimental configurations cannot be attributed to inconsistent ego-vehicle dynamics.

**Figure 5:**
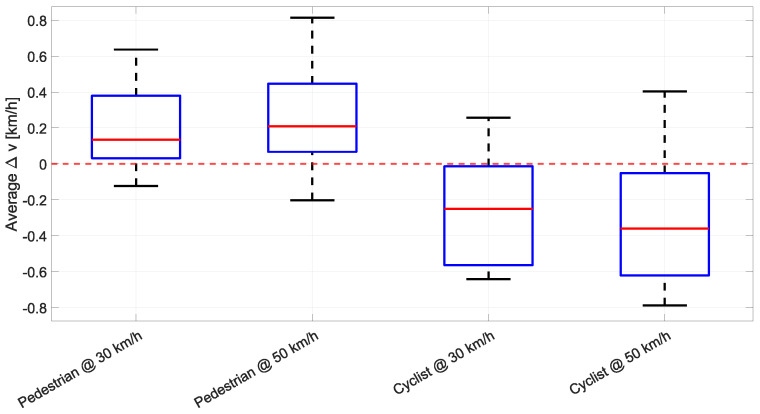
Target crossing velocity deviations (Average Δv) measured via radar for pedestrian and cyclist dummies at both ego-vehicle speeds. In the box plots, the solid red line indicates the median, the blue box the quartiles, black dashed lines the extreme values, and the horizontal red dashed line the zero reference.

Verification of target motion consistency was conducted through radar-based radial velocity measurements obtained via Doppler analysis. The radial velocity component measured by the radar represents the vector resultant of ego-vehicle longitudinal motion and target crossing motion, capturing the relative approach dynamics relevant for the detection performance evaluation [30]. Figure 5 presents the deviations in measured radial velocity from target values across all test scenarios. For pedestrian crossing scenarios, mean radial velocity deviations of 0.18 km/h and 0.20 km/h were observed for 30 km/h and 50 km/h ego-speed conditions respectively. The maximum observed deviations reached 0.64 km/h and 0.81 km/h, demonstrating tight consistency in relative motion dynamics across both speed conditions. For cyclist crossing scenarios, mean radial velocity deviations of negative 0.25 km/h and negative 0.32 km/h were observed for the respective ego-speed conditions, with maximum absolute deviations of 0.64 km/h and 0.79 km/h. It is notable that the negative mean deviations indicate a small systematic offset to lower speeds in cyclist crossing execution.

Examination of the data reveals that radial velocity deviations remain remarkably small across all test conditions, with mean deviations below 0.32 km/h and maximum deviations below 0.81 km/h for all scenarios. This consistency indicates that the relative motion dynamics between ego-vehicle and crossing target maintain stable execution characteristics across the tested velocity range, demonstrating robust scenario control throughout the measurement campaign. The consistent positive deviation for pedestrian scenarios and negative deviation for cyclist scenarios indicate systematic rather than random trajectory execution characteristics, further supporting comparability across measurement repetitions. Statistical analysis of vehicle telemetry across measurement campaigns revealed no systematic drift in experimental conditions over time, confirming that environmental factors and equipment calibration remained stable throughout data collection. These findings establish that the combination of ego-speed variability below 0.2 km/h and absolute radial velocity mean deviations below 0.32 km/h enables confident attribution of observed detection performance differences to headlight parameters and ambient lighting conditions rather than inconsistent scenario execution. With maximum radial velocity deviations remaining below 0.81 km/h across all test conditions, the consistency in relative motion dynamics between ego-vehicle and crossing targets provides a robust foundation for systematic parameter comparison in the subsequent analyses.

#### Overall Performance and Parameter Space Overview

**Figure 6:**
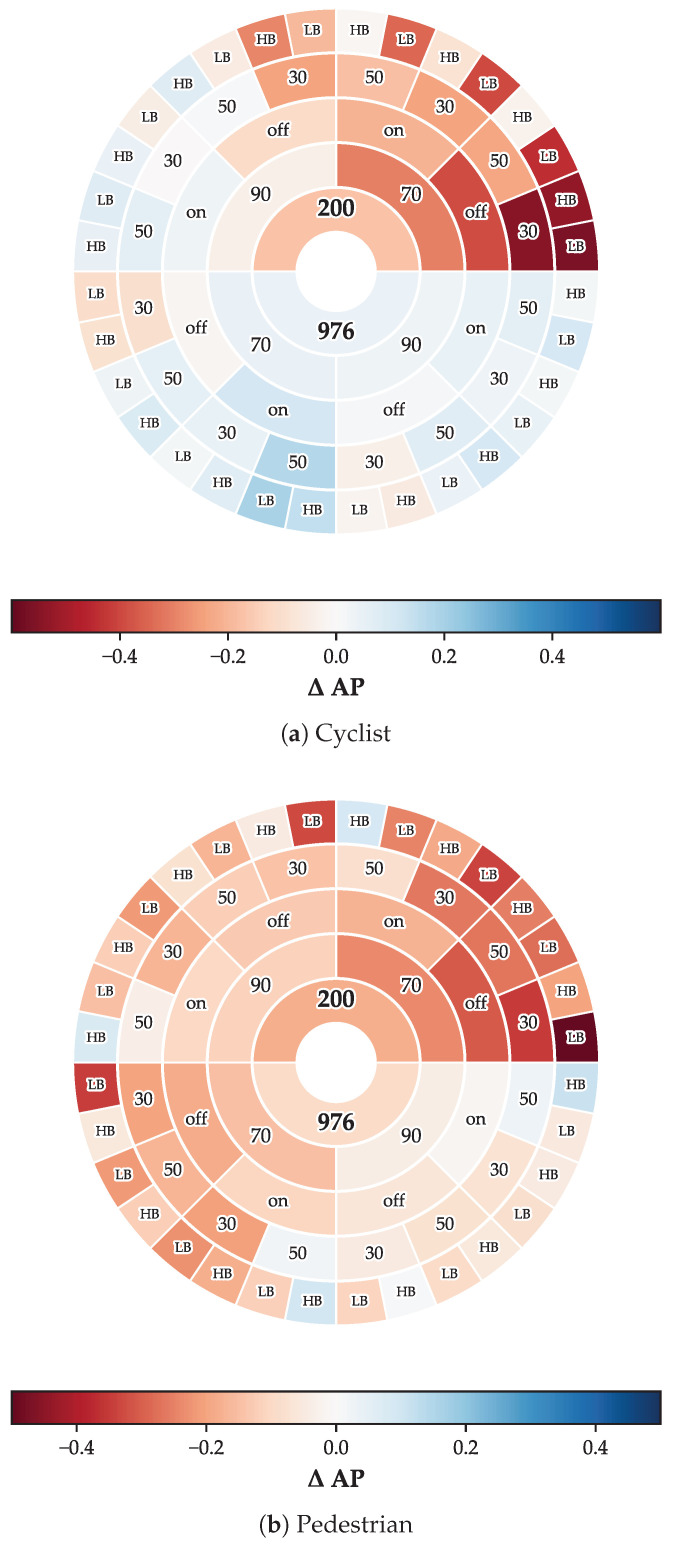
Sunburst visualization of parameter influence on ΔAP (Average Precision improvement over reference). Ring hierarchy from inner to outer: (1) PWM Frequency [Hz], (2) Duty Cycle [%], (3) Street Lighting [-], (4) Ego Velocity [km/h], (5) Headlight Setting [-]. Blue segments indicate positive ΔAP (improvement), red segments indicate negative ΔAP (degradation). (**a**) Cyclist detection scenario. (**b**) Pedestrian detection scenario.

Detection performance is quantified using AP at an intersection-over-union threshold of 0.5, consistent with standard object detection evaluation protocols [31]. Performance improvement or degradation relative to continuous illumination baseline is quantified as ΔAP, computed as the difference between modulated illumination AP and continuous illumination AP for each configuration, enabling direct assessment of when artificial event generation provides detection advantages versus disadvantages. The comprehensive parameter space defined by PWM frequency, duty cycle, street lighting conditions, ego-vehicle speed, and light distribution was characterized through 64 measurements spanning all factorial combinations for both cyclist and pedestrian targets. Figure 6 presents the complete parameter space through sunburst visualizations that encode both parameter combinations and their corresponding detection performance relative to the baseline scenario. The sunburst ring hierarchy organizes parameters from PWM frequency in the center through duty cycle, street lighting, ego-vehicle speed, to headlight distribution at the periphery, enabling identification of beneficial parameter combinations through color clustering where blue segments indicate improvement over baseline and red segments indicate degradation.

Cyclist detection AP values range from 0.086 to 0.872 across the evaluated parameter space, demonstrating that illumination configuration exerts a substantial influence on detection capability. The ΔAP distribution relative to continuous illumination baseline ranges from −0.564 to +0.193, indicating that certain PWM configurations provide AP improvements exceeding 19 percentage points while others introduce degradation exceeding 56 percentage points. Analysis of the 32 parameter combinations reveals that 56.2% achieve positive performance improvement over baseline continuous illumination, establishing that PWM-enhanced detection provides net benefit when parameters are appropriately selected. The three best-performing cyclist configurations employ a 976 Hz PWM frequency with a 70% duty cycle, achieving ΔAP values of +0.193, +0.167, and +0.149.

Pedestrian detection presents a substantially greater challenge than cyclist scenarios, as reflected in both absolute AP values and sensitivity to parameter selection. The AP range spans from 0.004 to 0.805, with ΔAP distribution ranging from −0.489 to +0.169. Only 15.6% of the 32 parameter combinations exceed baseline performance, indicating that the majority of parameter configurations degrade detection performance rather than improve it for lower-reflectivity targets. While bias parameter optimization improves absolute AP values for numerous pedestrian configurations, this tuning primarily reduces the magnitude of performance degradation rather than enabling additional configurations to exceed baseline continuous illumination performance. The disparity in positive configuration rates between cyclist detection (56.2%) and pedestrian detection (15.6%) underscores the critical importance of retroreflective characteristics for robust PWM-enhanced detection. Pedestrian targets lacking bicycle-grade retroreflective materials generate fewer artificial events under PWM illumination, reducing the signal-to-background contrast enhancement that enables improved detection when retroreflectors are present.

Statistical comparisons across object classes reveal a consistent performance gap favoring cyclist detection across all evaluated conditions. Examination of the sunburst visualization confirms that configurations yielding positive cyclist ΔAP frequently correspond to negative or marginal pedestrian ΔAP, illustrating that parameter optimization for one target class does not guarantee equivalent benefit for the other. The 40.6% difference in positive configuration rates between cyclist and pedestrian detection reflects fundamental differences in retroreflective surface area between bicycles equipped with regulatory reflectors and pedestrians wearing typical nighttime attire with limited or absent retroreflective elements.

The analysis shows that PWM-enhanced detection capability depends on the simultaneous optimization of multiple interacting factors, including modulation frequency, duty cycle, light distribution, vehicle dynamics, and street lighting conditions. In the following section we will examine specific parameter combinations and failure modes in detail to characterize the physical mechanisms governing detection performance and identify practical deployment recommendations for real-world autonomous driving systems.

### 4.2. Optimal and Suboptimal Parameter Combinations

Systematic examination of detection performance across the 64 evaluated parameter combinations reveals distinct success and failure regimes, characterized by specific parameter interactions. Analysis focuses on the five best-performing and five worst-performing configurations for each target class, providing a symmetric comparison of successful versus unsuccessful parameter selections. This approach identifies critical configurations representing the top and bottom for both cyclist and pedestrian detection, enabling characterization of the parameter interactions that govern detection success versus severe performance degradation.

#### 4.2.1. Best-Performing Configurations

The top-performing parameter sets, representing the five best cases for both cyclist and pedestrian targets, are summarized in Table 1. The five best-performing cyclist configurations achieve ΔAP ranging from +0.193 to +0.105, demonstrating a strong preference for the 976 Hz PWM frequency, which appears in 5 of 5 top cases. The duty cycle distribution shows 70% in 4 of 5 cases, while the ego-vehicle speed of 50 km/h appears in 4 of 5 configurations. The single best configuration employs a low beam distribution at 976 Hz with a 70% duty cycle, operating at 50 km/h under street lighting-enabled conditions, achieving a ΔAP of +0.193 and an absolute AP of 0.726, compared to the baseline AP of 0.532. At a frequency of 976 Hz, the 10 ms accumulation window captures approximately 10 complete PWM cycles, generating roughly 20 brightness transitions per pseudo-frame between ON and OFF states. The second-ranked configuration achieves a ΔAP of +0.167 with a high beam distribution at 976 Hz and a 70% duty cycle at 50 km/h without street lighting, demonstrating that it enables substantial improvements even without ambient illumination.

The critical enabling factor for cyclist detection success under both light distributions is the bicycle retroreflector’s characteristics and spatial positioning. Regulatory bicycle reflectors meeting ECE R88 specifications provide a retroreflection coefficient $R_{A}$ exceeding 20 candela per lux at typical automotive observation angles [23]. Photometric measurements at a 30 m distance reveal that bicycle tire retroreflectors produce a luminance of 42.92 cd/m^2^ compared to 1.76 cd/m^2^ from pedestrian torso clothing, representing approximately 24-fold brightness enhancement under identical headlight illumination conditions. The wheel-mounted design positions high-retroreflectivity elements near the road surface, where they receive direct illumination from the low and high beam setting. This ensures robust event generation regardless of the active light distribution. In addition, the retroreflector optimally reflects the light back and guarantees reliable detection of PWM-induced events. The distinctive circular geometry of wheel reflectors creates readily identifiable spatial patterns that support the extraction of network features. This combination of regulatory-compliant retroreflectivity that substantially exceeds diffuse surface characteristics, favorable spatial positioning within illuminated zones, and distinctive geometric features enables cyclist detection to succeed across diverse parameter combinations when the temporal sampling frequency remains adequate.

For pedestrian detection, the five best-performing configurations achieve ΔAP values ranging from +0.169 to +0.057, demonstrating that specific parameter combinations are required. All 4 configurations exceeding ΔAP of +0.10 employ a high beam distribution at a 50 km/h ego-vehicle speed with street lighting enabled. The single best configuration achieves a ΔAP of +0.169 with a high beam at 976 Hz and a 90% duty cycle. Notably, both 976 Hz and 200 Hz appear in the top configurations, and both 70% and 90% duty cycles are present, demonstrating that pedestrian detection success depends primarily on light distribution and ambient lighting rather than modulation parameters. The universal requirement for high beam in top-performing pedestrian configurations stems from the 24-fold lower luminance of pedestrian clothing compared to bicycle tire retroreflectors. The high beam distribution provides substantially greater photon flux to compensate for reduced surface reflectivity, delivering illumination intensity sufficient to generate brightness transitions exceeding event camera contrast thresholds, even on diffusely reflecting fabric surfaces.

The consistent requirement for street lighting in high-performing pedestrian configurations reflects spatially selective event generation under combined illumination sources. When street lighting provides uniform ambient brightness across the scene, PWM headlight illumination generates temporally varying brightness primarily on surfaces receiving direct headlight flux rather than uniformly across all illuminated regions. The stable ambient background brightness establishes a baseline illumination level against which PWM-induced brightness transitions on pedestrian targets create enhanced temporal contrast. This spatially selective event generation concentrates artificial events within target bounding boxes while limiting increases in background event density. As a result, it effectively improves signal-to-noise ratio beyond what passive surface reflection achieves under continuous illumination. The modest absolute pedestrian detection performance under optimal configurations, with best-case AP of 0.411 occurring exclusively during street lighting enabled conditions, reflects elevated background noise event rates induced by ambient illumination. Street lighting increases false positive contrast detection across the entire sensor array, degrading signal-to-noise ratios that constrain detection capability despite achieving positive ΔAP through PWM-enhanced temporal contrast on pedestrian targets. Under continuous headlight illumination with street lighting present, pedestrian targets become visually indistinguishable from ambient brightness levels and merge into background noise, whereas PWM modulation enhances target visibility through temporal brightness transitions that exceed the static ambient illumination.

The observation that 50 km/h ego-vehicle speed appears exclusively in the four top-performing pedestrian configurations exceeding ΔAP of +0.10, while 30 km/h appears predominantly in bottom-performing cases, suggests velocity-dependent detection enhancement. At higher approach speeds corresponding to 13.9 m/s, increased relative motion between the ego-vehicle and crossing target generates additional motion-induced brightness change events beyond those produced purely by PWM modulation. This motion-induced event contribution supplements PWM-induced events, improving overall signal density within accumulated pseudo-frames and enhancing signal-to-noise ratio, particularly for pedestrian targets where signal events from diffuse surface illumination remain marginal even under optimal PWM parameters.

#### 4.2.2. Suboptimal Configurations

Table 2 summarizes the parameter sets yielding the five lowest performance scores for both cyclist and pedestrian targets. The five worst performing cyclist configurations produce ΔAP ranging from −0.564 to −0.292, with the 200 Hz PWM frequency appearing in all 5 bottom cases in Table 2. However, detailed examination reveals that frequency alone does not determine failure, as 200 Hz configurations with a high beam and 90% duty cycle achieve positive performance with ΔAP values of +0.079 and +0.056. Severe degradation cases specifically combine 200 Hz with a low beam distribution and a 70% duty cycle, producing a mean ΔAP of −0.437 across all lighting and speed conditions. The worst single configuration employs a low beam distribution with a modulation frequency of 200 Hz and 70% duty cycle, 30 km/h ego-speed, and street lighting disabled, yielding ΔAP of −0.564, with the absolute AP dropping to 0.128 from the baseline 0.692, representing 81% performance degradation, where detection capability essentially collapses.

The physical mechanism underlying this substantial performance decline involves interaction between temporal sampling sparsity and background noise accumulation. At a 200 Hz frequency with 70% duty cycle, each PWM period spans 5.0 ms, comprising 3.5 ms ON and 1.5 ms OFF. The extended OFF duration allows substantial accumulation of background noise events as the sensor adapts to darker conditions during each cycle. Additionally, prolonged state durations yield larger brightness contrast magnitudes, which support the generation of noise events. The analysis of event camera contrast detection characteristics shows that background event generation rate increases at low illumination levels, according to sensor characterization measurements provided by Prophesee [32]. During the 1.5 ms OFF periods at 200 Hz with 70% duty cycle, the sensor sensitivity increases as brightness levels drop, generating noise events across the image that dilute signal events from target brightness transitions. Simultaneously, the 10 ms accumulation window captures only two complete PWM cycles at 200 Hz, yielding only four brightness transitions per pseudo-frame. This sparse temporal sampling combined with elevated background noise produces signal-to-noise ratios insufficient for reliable feature extraction by the detection network. The 90% duty cycle at 200 Hz mitigates this failure mode by reducing the OFF duration to 0.5 ms, limiting noise accumulation while maintaining near-continuous illumination that approaches baseline performance.

The five worst-performing pedestrian configurations produce ΔAP ranging from −0.489 to −0.315, demonstrating an exclusive association with low beam distribution, which appears in all 5 bottom cases, all at 30 km/h ego-speed. The worst configuration combines low beam with 200 Hz at 70% duty cycle, 30 km/h, and street lighting disabled, producing a ΔAP of −0.489, with the absolute AP collapsing to 0.011, representing near-complete detection failure. Examination across all 16 low beam pedestrian configurations reveals that none achieve a positive ΔAP, with the least negative case reaching only −0.025. This universal failure of low beam configurations stems from ECE photometric regulations that require horizontal cutoff characteristics for low beam distributions to prevent glare for oncoming traffic. The cutoff limits vertical illumination so that pedestrian torso and head regions receive substantially reduced illumination intensity compared to the road surface. For bicycle targets, wheel-mounted reflectors positioned near road level receive adequate low beam illumination, but pedestrian body regions above the cutoff boundary receive illumination intensities below thresholds required for event generation on diffusely reflecting clothing surfaces. The combination of a photometric cutoff, limiting illumination intensity, and diffuse surface characteristics that prevent strong brightness transitions results in insufficient event generation to support detection.

#### 4.2.3. Cross-Target Performance Comparison

Comparison of critical configurations across target classes reveals fundamentally different parameter sensitivity profiles that preclude universal optimization. Cyclist detection demonstrates strong frequency dependence, with 976 Hz enabling high performance across all top configurations. In contrast, 200 Hz produces severe degradation when combined with a low beam and 70% duty cycle, although it remains effective under other parameter selections. Pedestrian detection requires an absolute headlight setting, with a high beam light distribution mandatory for achieving positive performance, and accommodates both 976 Hz and 200 Hz frequencies when combined with a high beam and street lighting. The duty cycle parameter exhibits context-dependent behavior: 70% appears in top-performing configurations at 976 Hz but dominates in poor-performing configurations at 200 Hz across both target classes. These divergent requirements indicate that configurations optimized for cyclist detection via 976 Hz selection and a 70% duty cycle do not guarantee pedestrian detection success without a high beam setting, whereas high beam configurations optimized for pedestrian visibility may introduce constraints that limit the optimization of cyclist detection. In general, the PWM system offers an advantage on reflective surfaces, but this advantage is limited on surfaces with small reflection coefficients.

The optimal universal configuration employs a high beam distribution at a 976 Hz PWM frequency with a 70% duty cycle, a 50 km/h ego-vehicle speed, and street lighting enabled, achieving ΔAP of +0.149 for cyclist detection and +0.132 for pedestrian detection, with an average ΔAP of +0.140. The remaining two dual-positive configurations utilize high beam at 50 km/h with street lighting enabled, employing either 200 Hz at 90% duty cycle yielding average ΔAP of +0.106, or 976 Hz at 90% duty cycle yielding average ΔAP of +0.096. This scarcity of universally beneficial configurations, representing 9.4% of the evaluated parameter space, underscores that PWM parameter selection involves fundamental trade-offs between cyclist and pedestrian detection performance, with optimization for one target class often at the expense of the other. Consolidated parameter recommendations for cyclist-optimized, pedestrian-optimized, and universal deployment configurations are provided in Appendix A.

## 5. Discussion

A comprehensive parameter-space evaluation across 64 combinations of PWM frequency, duty cycle, light distribution, ego-vehicle speed, and ambient lighting, under Euro NCAP crossing-inspired scenarios, establishes fundamentally divergent optimization requirements for cyclist versus pedestrian detection. Cyclist detection achieves robust performance with a 976 Hz modulation frequency, tolerating both low beam and high beam distributions when combined with appropriate duty cycle selection, whereas a 200 Hz frequency produces severe degradation exclusively when paired with a low beam and a 70% duty cycle. Pedestrian detection requires high beam distribution in conjunction with street lighting to achieve performance improvements, whereas low beam configurations universally fail to exceed the continuous illumination baseline regardless of modulation parameters. Three of 32 evaluated parameter combinations provide simultaneous performance improvements for both target classes, establishing that universal optimization remains elusive and deployment scenarios require target-specific parameter selection or acceptance of suboptimal performance on at least one target class.

The limited number of universally beneficial parameter combinations presents significant challenges for mixed-traffic deployment scenarios where both cyclists and pedestrians may appear simultaneously. Among the three configurations achieving positive performance for both targets, high beam operation at 976 Hz with a 70% duty cycle, 50 km/h ego-speed, and street lighting enabled provides the most robust solution, with an average performance improvement of ΔAP = +0.140 across both target classes. However, practical deployment must account for regulatory constraints on high beam use in populated areas and the presence of oncoming traffic, necessitating target-specific optimization strategies tailored to operational context and risk assessment priorities. For applications where pedestrian safety takes precedence, conservative parameter selection mandates a high beam distribution for street lighting conditions and a minimum approach speed of 50 km/h, accommodating both 976 Hz and 200 Hz modulation frequencies with either 70% or 90% duty cycle. This configuration space provides redundancy against individual parameter variations while maintaining positive pedestrian detection performance. Cyclist detection under these parameters achieves acceptable though not optimal performance, representing a safety-oriented trade-off that prioritizes the more vulnerable road user class [33]. Conversely, cyclist-optimized deployments benefit from 976 Hz frequency with 70% duty cycle across both light distributions, enabling robust detection under diverse lighting conditions. However, this optimization provides minimal pedestrian detection capability under low beam operation, requiring operational awareness of reduced performance for non-retroreflective targets. The cost differential and reduced efficiency of 976 Hz compared to 200 Hz-capable LED driver electronics motivate the consideration of lower-frequency systems; yet, results demonstrate that 200 Hz operation demands careful parameter constraints to achieve a high beam with 90% duty cycle, substantially limiting operational flexibility compared to 976 Hz systems. Critical to deployment reliability, the system must incorporate fallback mechanisms that detect suboptimal operating conditions and revert to continuous illumination without PWM when parameter combinations enter degradation regimes. Monitoring of detection confidence scores, combined with parameter-state awareness, enables real-time switching between PWM-enhanced and continuous operation modes, ensuring that the enhancement system never degrades performance below conventional baseline capabilities. This fail-safe approach is particularly important during transitions between street lighting conditions or changes in light distribution, where momentary mismatches in parameters could introduce detection gaps.

The dynamic Euro NCAP-inspired crossing measurement validation presented in this work provides complementary insights to semi-static optimization results previously established at the HELLA testing environment [10]. Semi-static measurements conducted with a stationary ego-vehicle and controlled target approach trajectories yielded generally higher absolute AP values than dynamic crossing scenarios, reflecting reduced scene complexity, consistent target distances, ambient lighting, and simplified background conditions. Despite these absolute performance differences, the relative parameter dependencies remain consistent across validation methodologies, with 976 Hz frequency demonstrating superiority over 200 Hz under equivalent conditions, and high beam distribution proving essential for pedestrian detection in both static and dynamic scenarios. Dynamic validation reveals velocity-dependent effects absent from semi-static testing, particularly the observation that 50 km/h ego-speed yields superior pedestrian detection compared to 30 km/h. This counterintuitive finding suggests that motion-induced brightness change events generated by higher relative velocities supplement PWM-induced events to improve overall signal density, an effect that cannot manifest under stationary ego-vehicle conditions. Additionally, dynamic scenarios introduce temporal variations in target illumination as crossing trajectories transition through headlight beam patterns, creating realistic, challenging conditions where PWM parameter robustness becomes critical for maintaining continuous detection throughout crossing maneuvers. The consistency of fundamental parameter dependencies across static and dynamic validation scenarios supports the generalizability of the optimization principles to diverse operational contexts. The requirement for high retroreflectivity to achieve substantial PWM-enhancement benefits, the superiority of high-frequency modulation over low-frequency operation, and the selection of high duty cycles selection, all demonstrate robust manifestation across testing methodologies. This consistency indicates that insights from controlled validation scenarios provide reliable guidance for real-world parameter selection for deployment, despite inevitable increases in environmental complexity.

Several methodological constraints limit the scope and generalizability of the presented results. The controlled experimental environment employed uniform LED street lighting without PWM modulation, avoiding potential interference effects that may arise in real-world scenarios where street lighting itself may employ PWM. Concurrent PWM modulation from multiple sources operating at potentially interfering frequencies could introduce beat frequency artifacts or aliasing effects not captured in the single-source illumination scenarios evaluated. Additionally, the nighttime validation was conducted under clear atmospheric conditions, without precipitation, fog, or airborne particulates (smog) that substantially affect both continuous and PWM illumination propagation and event generation characteristics.

The detection network employed in this evaluation was trained exclusively on the DSEC dataset acquired under natural lighting conditions without artificial PWM, introducing potential domain mismatch between training and deployment conditions. This mismatch presents fundamental interpretational challenges: observed performance degradations under suboptimal PWM parameters may reflect either (1) insufficient event generation from inappropriate illumination parameters, or (2) network feature extractors optimized for natural brightness transitions failing to exploit artificial PWM-induced temporal patterns effectively. The relative performance comparisons (ΔAP) between parameter configurations remain robust to network training bias, as all measurements employ identical detection architecture and training procedure, enabling valid identification of optimal versus suboptimal parameter selections within the evaluated space. However, absolute AP values fundamentally depend on the sensor–network system pair and cannot be generalized to alternative detection architectures or training procedures without additional validation. Networks specifically trained on PWM-enhanced event datasets or employing temporal feature extraction mechanisms designed to exploit artificial brightness transitions may achieve substantially different absolute performance levels while maintaining similar relative parameter dependencies. The recent release of nighttime-specific event camera datasets incorporating artificial illumination scenarios may enable future network training specifically optimized for PWM-enhanced detection [34]. Such specialized training could potentially alter the relative performance characteristics observed with networks trained on conventional datasets. The feature extraction mechanisms learned during DSEC training may not optimally exploit the artificial brightness transitions introduced by PWM modulation, suggesting that purpose-trained networks could achieve superior performance under identical parameter configurations.

Validation employed a single event camera sensor model and detection network architecture, limiting conclusions about sensor-agnostic or network-agnostic optimization principles. Different event camera implementations with varying contrast sensitivity thresholds, pixel response characteristics, and bias tuning ranges may exhibit different optimal parameter combinations. Similarly, detection networks with alternative temporal aggregation mechanisms, feature extraction architectures, or training datasets would likely demonstrate different parameter dependencies. Therefore, the presented results reflect the optimization of a specific sensor-network configuration, rather than universal principles applicable to all event-based detection implementations. Target variability limitations constrain the understanding of performance robustness across real-world diversity. Each measurement scenario contained a single isolated target without occlusions, background clutter, or simultaneous multiple target instances. Real-world mixed-traffic scenarios involving concurrent detection of multiple cyclists, pedestrians, and other road users with varying retroreflective properties and clothing characteristics introduce complexity not addressed in the controlled crossing scenarios. Additionally, pedestrian clothing retroreflectivity exhibits substantial variability across seasons, weather conditions, and cultural contexts, while the evaluation employed limited representative samples that may not capture the full range of real-world surface reflectance characteristics.

Multiple research directions emerge from the presented validation results to address identified limitations and extend PWM-enhanced detection capabilities. Adaptive parameter tuning mechanisms that dynamically adjust PWM frequency, duty cycle, or bias settings based on real-time scene analysis and detection confidence metrics could optimize performance across varying operational conditions without requiring manual parameter selection. Machine learning approaches that predict optimal parameter combinations from ambient lighting measurements, target class distributions, or historical detection performance could enable intelligent parameter adaptation that maximizes detection capability under current conditions while avoiding degradation regimes [9,10]. Multi-objective optimization network incorporating diverse road user classes beyond cyclists and pedestrians would address practical mixed-traffic scenarios more comprehensively. Future evaluations should be extended to the detection of other vulnerable road users and vehicles at close range, as well as road infrastructure elements, such as traffic signs and lane markings, under PWM illumination. Understanding parameter effects on broader object classes enables holistic system optimization that balances detection performance across all safety-relevant scene elements rather than optimizing for isolated target classes. The parameter space providing simultaneous improvement for both target types constrains mixed-traffic deployment strategies. Three of thirty-two tested configurations achieve positive detection enhancement for both cyclists and pedestrians. Three complementary approaches offer practical implementation paths. Context-aware static parameter selection deploys fixed configurations determined by operational context and regulatory constraints. Urban environments with mixed pedestrian and cyclist traffic face legal restrictions on high beam usage under ECE regulations, constraining deployment to low beam configurations that favor cyclist detection [26]. Rural environments permit the compromise parameter configuration achieving modest improvement for both target types, though this requires acceptance of suboptimal performance compared to target-specific configurations. In scenarios where available parameter combinations yield marginal improvement or risk detection degradation, fallback to continuous illumination without PWM modulation represents a conservative strategy ensuring baseline detection performance. This approach requires no real-time switching hardware and leverages existing vehicle positioning systems for regulatory-compliant configuration selection. Spatially selective illumination with high-resolution matrix LED technology represents the most promising solution, enabling simultaneous deployment of target-specific PWM parameters across different spatial regions within a single frame [35]. Modern pixel light modules with 25,000+ individually controllable segments permit cyclist-optimized parameters at road level while pedestrian-optimized parameters illuminate vertical zones, circumventing temporal switching limitations and aligning with automotive lighting evolution toward adaptive high-resolution systems. Adaptive temporal switching monitors detection confidence outputs to adapt parameters for subsequent frames based on identified target class composition, though this introduces latency between target appearance and optimal parameter activation. For near-term deployment, context-aware selection offers immediate viability with conventional headlights, while spatial segmentation with matrix LED technology provides the preferred long-term solution for advanced lighting platforms. These approaches can be combined based on available hardware capabilities. Repeated trial validation incorporating formal statistical testing would further strengthen the quantitative robustness of the reported parameter dependencies. While the current single-measurement characterization across 64 configurations benefits from strictly controlled conditions validated through consistency analysis, conducting multiple trials per configuration would yield formal confidence intervals for critical performance comparisons. Particular focus should be directed toward the binary failure modes identified in this work, specifically the universal low beam pedestrian detection failure and the severe cyclist degradation observed under the 200 Hz, low beam, and 70% duty cycle conditions. Statistical validation would confirm the robustness of these failure modes and establish confidence in the generalizability of the identified degradation regimes. Real-world field validation under adverse weather conditions, oncoming vehicle interactions, and diverse PWM-LED source environments would establish performance robustness beyond controlled laboratory scenarios. Precipitation introduces complex light-scattering and reflection phenomena that substantially alter both the illumination distribution and the event-generation characteristics. Oncoming vehicle headlights create dynamic illumination interference that may enhance or degrade detection depending on temporal modulation synchronization. Urban environments increasingly employ approaching vehicles with PWM headlights, PWM-controlled LED street lighting and advertising displays that introduce multiple concurrent modulation sources operating at various frequencies, creating potential interference or enhancement effects requiring systematic characterization. Extended sensor and network validation across alternative event camera platforms with varying pixel architectures, dynamic ranges, and temporal resolutions would establish the generalizability of optimization principles beyond the Prophesee IMX636 platform used in this work. Similarly, evaluation of detection networks specifically trained on nighttime datasets or purpose-designed for PWM-illuminated scenarios would determine whether network architecture adaptations can further improve performance beyond what sensor-level parameter optimization achieves. Investigation of novel network architectures that explicitly model PWM temporal patterns or incorporate frequency-domain analysis of brightness transition characteristics may unlock performance improvements beyond those achieved by conventional detection networks when applied to PWM-enhanced event streams [6].

## 6. Conclusions

This study systematically evaluated PWM-enhanced event-based detection across 64 parameter combinations under Euro NCAP-inspired crossing scenarios to establish optimization requirements for nighttime vulnerable road user detection. Parameter space analysis revealed performance ranging from substantial improvements (ΔAP = +0.193) to severe degradation (ΔAP = −0.564) relative to the continuous illumination baseline, demonstrating that parameter selection critically determines whether enhancement or degradation occurs. Results show that optimal PWM parameter configurations maintain competitive detection performance at a 10 ms accumulation time, compared to the 50 ms baseline used during network training, representing a five-fold reduction in processing latency critical for automotive safety applications. Cyclist detection achieves robust performance, with 976 Hz modulation frequency appearing in all five top-performing configurations. In contrast, 200 Hz operation combined with low beam distribution and 70% duty cycle produces mean ΔAP of −0.437 due to background noise accumulation during extended OFF periods of the headlight. Pedestrian detection requires high beam distribution with street lighting enabled; low beam configurations universally fail to exceed the continuous illumination baseline across all 16 evaluated combinations. Three of thirty-two parameter combinations achieve simultaneous improvements for both target classes. PWM-enhanced detection performs optimally on highly retroreflective surfaces that meet ECE R88 specifications, whereas diffusely reflecting pedestrian clothing fundamentally limits detection capability despite parameter optimization. Deployment requires target-specific parameter selection strategies, though universal configurations using high beam at 976 Hz with 70% duty cycle are viable for mixed-traffic scenarios under street lighting conditions. Future work should address adaptive parameter tuning in response to real-time operational conditions, repeated-trial statistical testing to establish formal significance levels, real-world field validation under adverse weather conditions, and extended sensor evaluation across alternative event camera platforms and detection architectures specifically trained on nighttime or PWM-illuminated scenarios.

## Figures and Tables

**Table 1:** Top 5 best-performing parameter configurations for cyclist and pedestrian detection. Configurations represent highest ΔAP values. HB: high beam, LB: low beam.

Target	Beam	Freq [Hz]	Duty [%]	Speed [km/h]	Street Light	ΔAP	AP
Cyclist	LB	976	70	50	ON	+0.193	0.726
Cyclist	HB	976	70	50	OFF	+0.167	0.872
Cyclist	HB	976	70	50	ON	+0.149	0.780
Cyclist	HB	200	70	30	ON	+0.140	0.697
Cyclist	LB	976	90	50	ON	+0.107	0.640
Pedestrian	HB	976	90	50	ON	+0.169	0.411
Pedestrian	HB	200	90	50	ON	+0.156	0.398
Pedestrian	HB	200	70	50	ON	+0.142	0.383
Pedestrian	HB	976	70	50	ON	+0.132	0.373
Pedestrian	HB	976	90	30	OFF	+0.057	0.786

**Table 2:** Bottom 5 worst performing parameter configurations for cyclist and pedestrian detection. Configurations represent the most severe performance degradation. HB: high beam, LB: low beam.

Target	Beam	Freq [Hz]	Duty [%]	Speed [km/h]	Street Light	ΔAP	AP
Cyclist	LB	200	70	30	OFF	−0.564	0.128
Cyclist	LB	200	70	50	OFF	−0.443	0.298
Cyclist	LB	200	70	30	ON	−0.394	0.086
Cyclist	LB	200	70	50	ON	−0.345	0.187
Cyclist	HB	200	90	30	OFF	−0.292	0.498
Pedestrian	LB	200	70	30	OFF	−0.489	0.011
Pedestrian	LB	976	70	30	OFF	−0.339	0.180
Pedestrian	LB	200	90	30	OFF	−0.331	0.189
Pedestrian	LB	200	70	30	ON	−0.315	0.004
Pedestrian	LB	200	70	50	OFF	−0.245	0.078

## Data Availability

The data that have been used are confidential.

## Abbreviations

The following abbreviations are used in this manuscript:

AP	Average Precision
CAN	Controller Area Network
DSEC	Stereo Event Camera Dataset for Driving Scenarios
ECE	Economic Commission for Europe
Euro NCAP	European New Car Assessment Programme
GPS	Global Positioning System
IMU	Inertial Measurement Unit
LED	Light Emitting Diode
PWM	Pulse Width Modulation
Radar	Radio Detection and Ranging
RCS	Radar Cross Section
RGB	Red Green Blue
SDK	Software Development Kit
USB	Universal Serial Bus
YOLO	You Only Look Once

## Appendix A. Recommended Parameter Ranges for Target-Specific Optimization and Mixed-Traffic Deployment

**Table A1:** Recommended parameter ranges for target-specific optimization and mixed-traffic deployment based on comprehensive evaluation across 32 parameter combinations under Euro NCAP crossing scenarios. Universal configuration achieves positive performance improvements for both target classes. Bold text indicates mandatory requirements for positive performance. HB: high beam, LB: low beam.

Parameter	Cyclist-Optimized	Pedestrian-Optimized	Universal
PWM Frequency	**976 Hz**	976 Hz or 200 Hz	**976 Hz**
Duty Cycle	**70%**	70–90%	**70%**
Beam Distribution	LB or HB	**HB only**	**HB**
Ego-Vehicle Speed	30–50 km/h	**50 km/h**	**50 km/h**
Street Lighting	OFF or ON	**ON**	**ON**
Expected ΔAP Range	+0.107 to +0.193	+0.057 to +0.169	+0.132 to +0.149
Best Configuration ΔAP	+0.193	+0.169	+0.149
Absolute AP Range	0.640 to 0.872	0.373 to 0.786	0.373 to 0.780
